# Supplementary material for: Pathogenic diversification of the gut commensal Providencia alcalifaciens via acquisition of a second type III secretion system
Source: Infect Immun. 2024 Sep 10;92(10):e00314-24. doi: 10.1128/iai.00314-24 (PMC11477908; doi:10.1128/iai.00314-24)
Supplement: Supplemental figures — Fig. S1-S4. [file iai.00314-24-s0001.pdf]

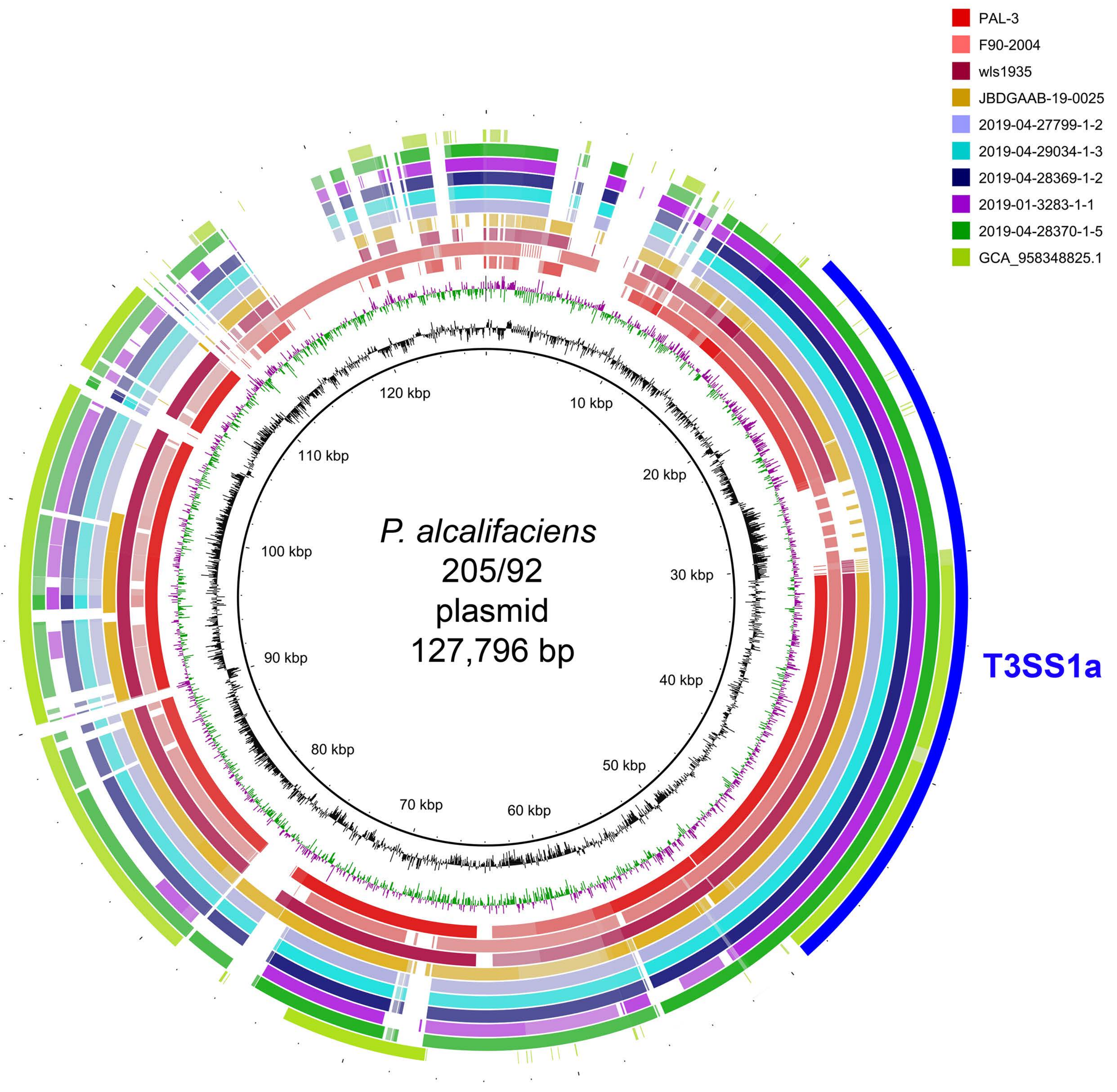

Figure S1: Alignment of p128kb. BRIG diagram of p128kb alignment from the ten *P. alcalifaciens* strains that harbor p128kb and are not shown in Figure 1B (PAL-3, F90-2004, wls1935, JBDGAAB-19-0025, 2019-04-27799-1-2, 2019-04-29034-1-3, 2019-04-28369-1-2, 2019-01-3283-1-1, 2019-04-28370-1-5, GCA_958348825.1). p128kb of *P. alcalifaciens* 205/92 presented in this work is used as a reference. Exact coordinates of T3SS1a are provided in Table S1.

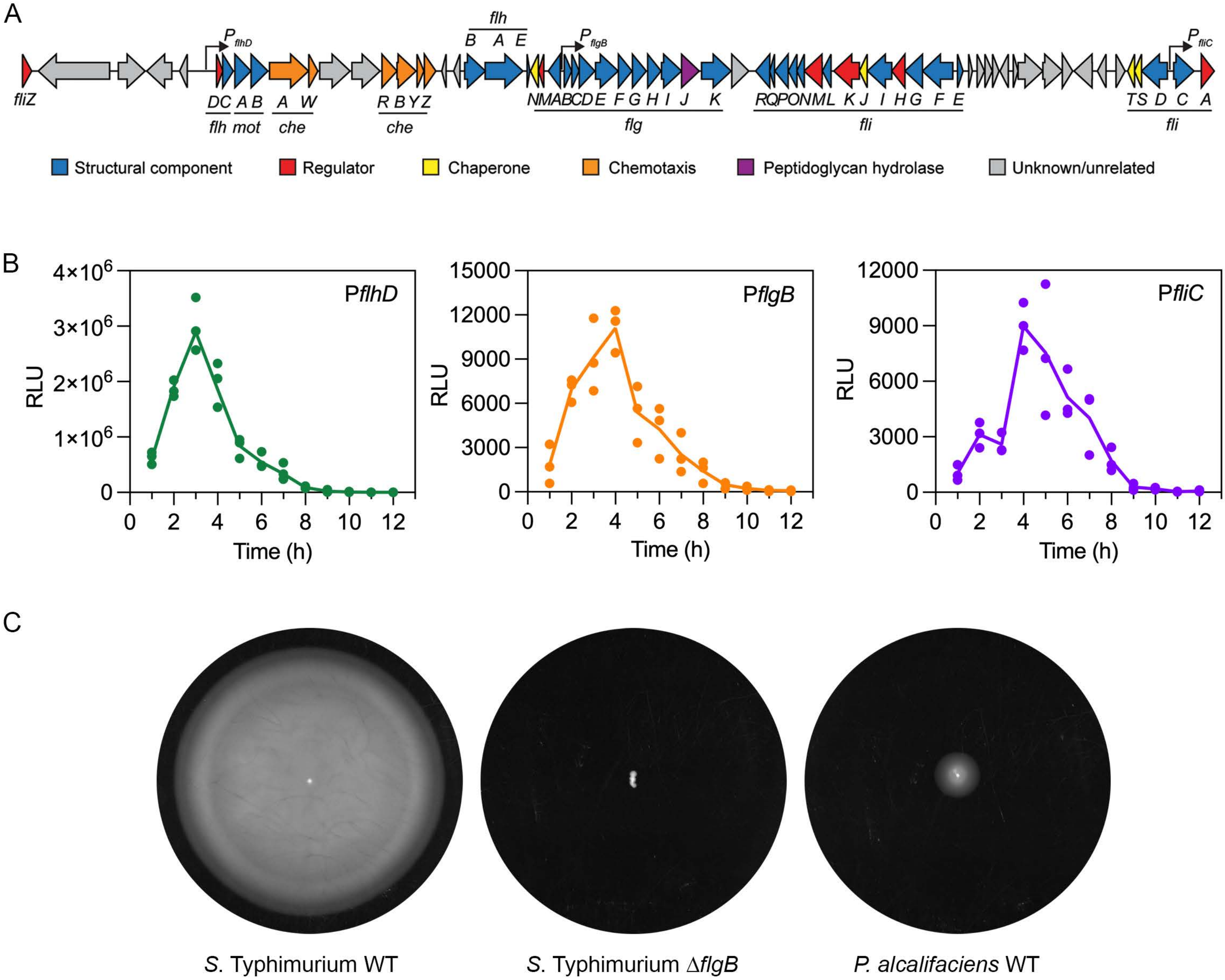

Figure S2: *P. alcalifaciens* is poorly motile. (A) Schematic of the *P. alcalifaciens* 205/92 genomic region containing flagella-associated genes. (B) Promoter activity of flagella regulators and structural components reported as 10e4 relative light units (RLUs). Data are from ≥3 independent experiments (mean ± SD). (C) Swimming motility of *P. alcalifaciens* compared to *S*. Typhimurium wild type (WT) and a non-motile *S*. Typhimurium Δ*flgB* mutant. Overnight bacterial cultures were inoculated into semi-solid LB agar plates and incubated overnight at 37°C.

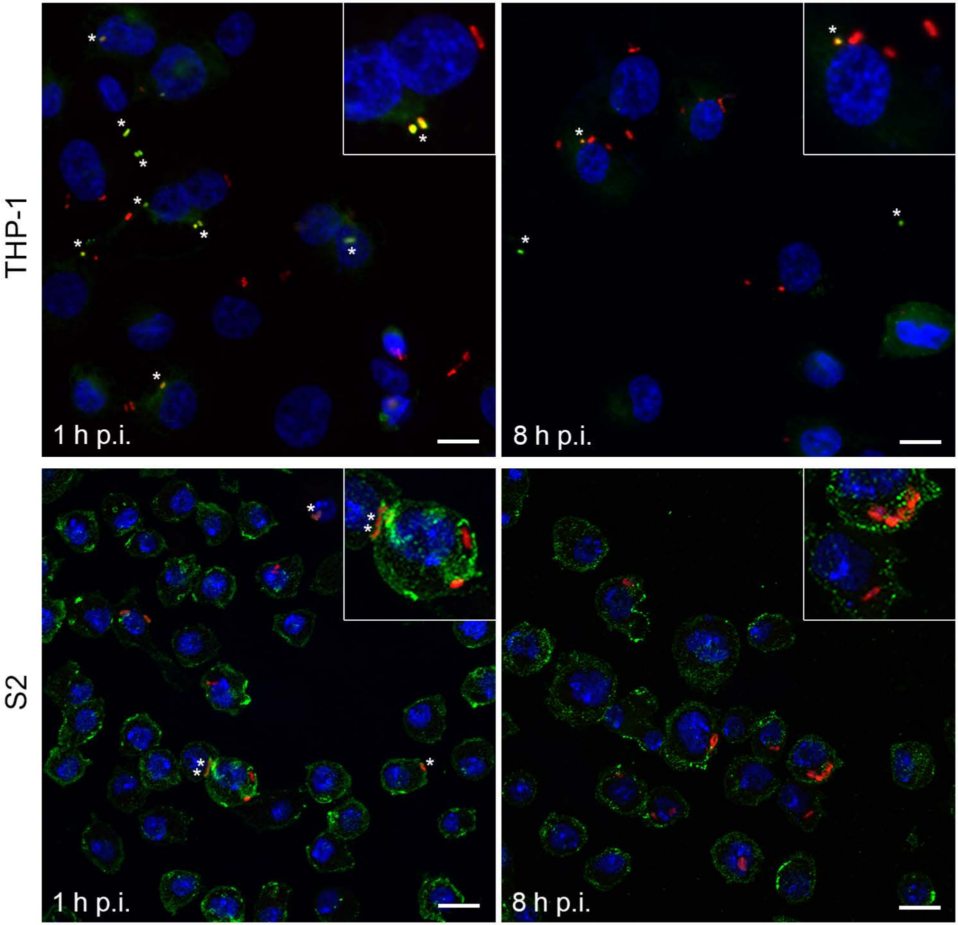

Figure S3: Inside-outside staining. Fluorescence staining without eukaryotic cell permeabilization allows for differentiation of extracellular and intracellular bacteria. Representative widefield and confocal fluorescence microscopy images of THP-1 and S2 cells, respectively, infected with *P. alcalifaciens* harboring pGEN-DsRed.T3. Cells were fixed at 1 h and 8 h p.i. and extracellular bacteria were detected with rabbit polyclonal anti-*P. alcalifaciens* antibodies, followed by anti-rabbit Alexa 488 antibodies. All bacteria fluoresce red (total) and extracellular bacteria fluoresce green (indicated by asterisks). Note that the anti-*P. alcalifaciens* antibodies cross-react with an S2 cell-surface protein(s). Scale bars are 10 µm.

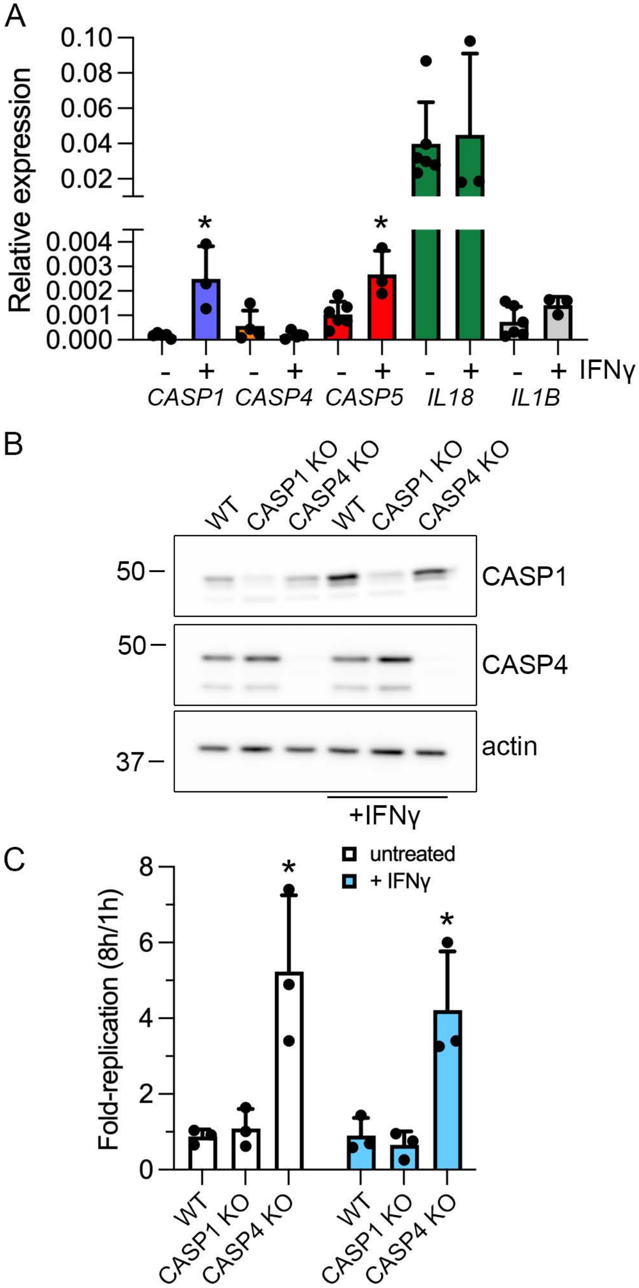

Figure S4: Caspase-4 restricts bacterial replication in C2Bbe1 cells without IFNg priming. (A) C2Bbe1 cells were left untreated or treated with 50 ng/ml IFNg for 16-18h. mRNA expression of *CASP1*, *CASP4*, *CASP5*, *IL18* and *IL1B* relative to the reference gene, *RPLP0*, was measured by qPCR (expressed as $2^{-\Delta Cq}$). n=3-6 independent experiments. Asterisks indicate data significantly different from untreated cells, p<0.05, Student's t-test.
(B) Immunoblot analysis of caspase-1, caspase-4 and actin (loading control) in C2Bbe1 WT, *CASP1*$^{-/-}$ (CASP1 KO) or *CASP4*$^{-/-}$ (CASP4 KO) cells left untreated or treated with IFNg for 16-18h. (C) Bacterial replication was assessed by gentamicin protection assay. Fold-replication is CFU_{8h}/CFU_{1h}. Asterisk indicates data significantly different from C2Bbe1 WT, p<0.05, ANOVA with Dunnett's post-hoc test. n=3 independent experiments.
