# Supplementary material for: Pathogenic diversification of the gut commensal Providencia alcalifaciens via acquisition of a second type III secretion system
Source: Infect Immun. 2024 Sep 10;92(10):e00314-24. doi: 10.1128/iai.00314-24 (PMC11477908; doi:10.1128/iai.00314-24)
Supplement: Table S2 — Oligos used for cloning. [file iai.00314-24-s0003.docx]

**Table S2: Oligonucleotides used for cloning**

| **Name** | **Sequence (5’ to 3’)** | **Description** |
| --- | --- | --- |
| PinvF1a-BamF | CGGGATCCTGAGTTTCAGTAAGATGGGAT | pFU35-*PinvF*_1a_*-luxCDABE* |
| PinvF1a-KpnR | GGGGTACCATGATTAAACTCAACGTAATT |  |
| PsicA1a-BamF | CGGGATCCTACTTCAGACAGGATTTATCACCA | pFU35-*PsicA*_1a_*-luxCDABE* |
| PsicA1a-KpnR | GGGGTACCAATAAATCTCCGTTTTTATATCGC |  |
| PprgH1a-BamF | CGGGATCCGATTTTGATAAGTTTAGGTCTG | pFU35-*PprgH_1a_-luxCDABE* |
| PprgH1a-KpnR | GGGGTACCCTCAAAATTAACTAGCTCCTC |  |
| PinvF1b-BamF | CGGGATCCCATGATGCGTCTTCCATTCATC | pFU35-*PinvF*_1b_*-luxCDABE* |
| PinvF1b-KpnR | GGGGTACCTATAAATCAGTCCTATTCATTTGG |  |
| PsicA1b-BamF | CGGGATCCCGACACATATTGCCATGTTGATTT | pFU35-*PsicA*_1b_*-luxCDABE* |
| PsicA1b-KpnR | GGGGTACCATTTTCACCTCGTTTATCATGCGT |  |
| PprgH1b-BamF | CGGGATCCCGGTGATGATATTTCATCATC | pFU35-*PprgH_1b_-luxCDABE* |
| PprgH1b -KpnR | GGGGTACCAAAGACAATCTCAATTAATAAAA |  |
| PflhD-BamF | CGGGATCCcttatggtgaatctaaatgttg | pFU35-*PflhD-luxCDABE* |
| PflhD-KpnR | GGGGTACCtgaatctgaaatcccgagtct |  |
| PflgB-BamF | CGGGATCCGTAGCAACATAGTATTGCCC | pFU35-*PflgB-luxCDABE* |
| PflgB-KpnR | GGGGTACCGGTTTCCTCAATGGATTTCAG |  |
| PfliC-BamF | CGGGATCCAAATGCACTGATTTGCGCATC | pFU35-*PfliC-luxCDABE* |
| PfliC-KpnR | GGGGTACCAATGTATTGTCCTTTTTATCTTA |  |
| Kpn-dPainvA1a-F | GGGGTACCGATGAATTATTACGACAAGC | Δ*invA*_1a_ |
| Xma-dPainvA1a-R | TCCCCCCGGGCTGTAACTAATGGTTCACTC |  |
| dPainvA1a-OLF | GGATAATTCATA**GTG**TTTATC**TAA**ATCAAGGTTAATTATTTA |  |
| dPainvA1a-OLR | ATTAACCTTGAT**TTA**GATAAA**CAC**TATGAATTATCCTGATAG |  |
| Kpn-dPainvA1b-F | ggggtaccgcagcaattacgtgcatttc | Δ*invA*_1b_ |
| Xma-dPainvA1b-R | TCCCCCCGGGcactggcggattatcaatac |  |
| dPainvA1b-OLF | ataaacgatttc**atg**aaaata**taa**ggaacgcgagaatgaaatacc |  |
| dPainvA1b-OLR | tcattctcgcgttcc**tta**tatttt**cat**gaaatcgtttatttccta |  |
| PinvF1a-lux-BamF | See above | pGEN-*P_invF1a_invA1a* |
| InvA1a-Xma-R | tccccccggg**tta**gattgtttttataacatt |  |
| PinvF1ainvA1a-F2 | ttataattacgttgagtttaatc**atg**tgtttaactccctgcttaatagt |  |
| PinvF1ainvA1a-R2 | Actattaagcagggagttaaaca**cat**gattaaactcaacgtaattataa |  |

Engineered restriction sites are underlined. Start/stop codons in **bold**.
